# Water stabilizes an alternate turn conformation in horse heart myoglobin

**DOI:** 10.1038/s41598-023-32821-z

**Published:** 2023-04-13

**Authors:** Alex Bronstein, Ailie Marx

**Affiliations:** Department of Computer Science, Technion–Israel Institute of Technology, Haifa, Israel

**Keywords:** Structural biology, Protein folding

## Abstract

Comparison of myoglobin structures reveals that protein isolated from horse heart consistently adopts an alternate turn conformation in comparison to its homologues. Analysis of hundreds of high-resolution structures discounts crystallization conditions or the surrounding amino acid protein environment as explaining this difference, that is also not captured by the AlphaFold prediction. Rather, a water molecule is identified as stabilizing the conformation in the horse heart structure, which immediately reverts to the whale conformation in molecular dynamics simulations excluding that structural water.

## Introduction

Sperm whale myoglobin was the first, high resolution, protein structure ever solved by X-ray diffraction analysis^1^. Today this small, globular, single-domain protein is used as a model in exploring protein folding^2^. Myoglobin is represented in the Protein Data Bank (PDB) by hundreds of structures. The large number of independent experimental realizations of this protein structure provides a unique opportunity to identify features intrinsic to the protein and not the result of specific experimental artifacts. Here we observe that horse heart myoglobin consistently adopts a unique loop conformation and show, using molecular dynamics, that a structural water is necessary and sufficient for maintaining this conformation. This unique conformation highlights a structural feature which state of the art structure prediction methods such as AlphaFold remain blind to.

## Results

**Figure 1:**
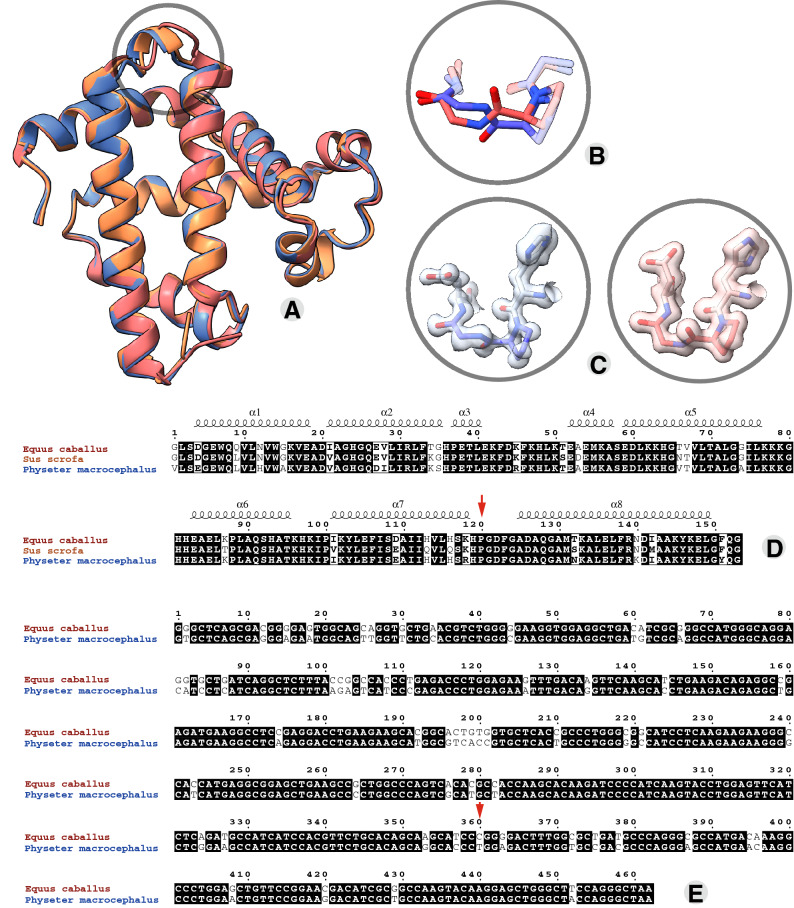
Horse heart myoglobin adopts an alternate turn conformation which is not explainable at the sequence level. *Eq_*myoglobin (1GJN, maroon), *Phy_*myoglobin (1A6K, blue) and *Sus_*myoglobin (1MWC, orange) structure alignment (**A**), highlighting the altered *Eq_*myoglobin turn conformation when the loops bridging helices G and H are locally aligned (**B**). This region of the protein chain is well-defined and distinct in the electron density maps (**C**). High identity is demonstrated in alignment of the amino acid sequence (**D**) and alignment of the coding sequences highlights synonymous differences (different coding sequences translating to the same amino acid), including on the loop itself (**E**). Alternate turn location is indicated with an arrow.

The hundreds of myoglobin structures available in the PDB share a very high degree of structural similarity across crystallization conditions, species, and mutations (Fig. 1A). None-the-less, variations exist, ranging from the major global conformational changes in domain swapped heterodimeric myoglobin to the local and specific adoption of different loop conformations in the interhelical region between helices G and H. This loop adopts a very different spatial orientation between well aligned helices in otherwise well aligned structures (Fig. 1A). When these loops are locally aligned it is observed that they have different turn conformations; Type I is adopted in the typical whale structure and Type II in the typical horse structure (Fig. 1B). The differences in this loop orientation was noted originally in the first high resolution structural comparison between sperm whale myoglobin (*Phy_*myoglobin) and horse heart myoglobin (*Eq_*myoglobin)^3^ and was attributed to an altered bonding network resulting from minor primary sequence differences. Many structures later, we suggest that sequence variations cannot clearly account for this difference. Evans and Bayer^3^ noted that the H12N substitution creates an N12–K16 interaction in *Eq_*myoglobin at the expense of the K16–D122 salt bridge in *Phy_*myoglobin, and also that the D27–R118 pair in *Eq_*myoglobin form a salt bridge not found between the equivalent E27–K118 pair in *Phy_*myoglobin. However, pig myoglobin (*Sus_*myoglobin), also characterized by tens of high-resolution structures, has the same turn conformation as *Phy_*myoglobin despite having the H12N and D27–R118 sequence features of *Eq_*myoglobin (Fig. 1D). Unique to *Eq_*myoglobin is Q9 (L9 in *Phy_*myoglobin, *Sus_*myoglobin and all homologues crystallized to date) that forms a polar interaction with D126 of helix H. However, we discount this feature as directly accounting for the altered conformation since there are *Eq_*myoglobin adopting the *Phy_*myoglobin conformation, described below, and without any alteration to or around the Q9 either in sequence or space.

Analysis of the structures reveals that the loop is structured in both horse and whale myoglobin, being defined by clear electron density (Fig. 1C) and typically having average to low B factors with respect to the rest of the structure. The G and H helices are well aligned between the horse and whale structures, whilst the loop bridging them occupies distinct positions, excluding the possibility of modeling ambiguity as explaining the different conformations. To track the source of the highly reproducible, alternate loop conformations we assessed all myoglobin structures. Table 1 shows that most species adopt the type I turn conformation, the clear exception being *Eq*_Myoglobin for which the type II turn is the preferred conformation. Analysis of these PDB entries verifies that crystallization conditions cannot readily explain the altered turn conformations; *Eq_*myoglobin and *Phy_*myoglobin are both represented by numerous structures crystallized in space groups P 2_1_ 2_1_ 2_1_ and P 1 2_1_ 1, and crystallized from conditions of both high salt concentration (usually ammonium sulphate) and various molecular weight polyethylene glycol (PEG) solutions.

**Table 1:** Categorization of the turn conformation adopted by myoglobin structures.

Entry	Species*^,#^	Typical conformation in whale myoglobin	Typical conformation in horse myoglobin
P02144	*Homo sapiens* (Human)	3RGK—dual conformation	3RGK—dual conformation
P02184	*Kogia sima* (Dwarf sperm whale)	6BMG	–
P02185	*Physeter macrocephalus* (Sperm whale)	101M, 102M, 103M, 104M, 106M, 107M, 108M, 109M, 110M, 111M, 112M, 1A6G, 1A6K, 1A6M, 1A6N, 1ABS, 1AJG, 1AJH, 1BVC, 1BVD, 1BZ6, 1BZP, 1BZR, 1CH1, 1CH2, 1CH3, 1CH5, 1CH7, 1CH9, 1CIK, 1CIO, 1CO8, 1CO9, 1CP0, 1CP5, 1CPW, 1CQ2, 1DO1, 1DO3, 1DO4, 1DO7, 1DTI, 1DTM, 1DUK, 1DUO, 1DXC, 1DXD, 1EBC, 1F63, 1F65, 1F6H, 1FCS, 1H1X, 1IOP, 1IRC, 1J3F, 1J52, 1JDO, 1JP6, 1JP8, 1JP9, 1JPB, 1JW8, 1L2K, 1LTW, 1LUE, 1MBC, 1MBD, 1MBI, 1MBN, 1MBO, 1MCY, 1MGN, 1MLF, 1MLG, 1MLH, 1MLJ, 1MLK, 1MLL, 1MLM, 1MLN, 1MLO, 1MLQ, 1MLR, 1MLS, 1MLU, 1MOA, 1MOB, 1MOC, 1MOD, 1MTI, 1MTJ, 1MTK, 1MYM, 1MYZ, 1MZ0, 1N9F, 1N9H, 1N9I, 1N9X, 1NAZ, 1O16, 1OBM, 1OFJ, 1OFK, 1SPE, 1SWM, 1TES, 1U7R, 1U7S, 1UFJ, 1UFP, 1V9Q, 1VXA, 1VXB, 1VXC, 1VXD, 1VXE, 1VXF, 1VXG, 1VXH, 1WVP, 1YOG, 1YOH, 1YOI, 2BLH, 2BLI, 2BLJ, 2BW9, 2BWH, 2CMM, 2D6C, 2E2Y, 2EB8, 2EB9, 2EF2, 2EKT, 2EKU, 2EVK, 2EVP, 2G0R, 2G0S, 2G0V, 2G0X, 2G0Z, 2G10, 2G11, 2G12, 2G14, 2JHO, 2MB5, 2MBW, 2MGA, 2MGB, 2MGC, 2MGD, 2MGE, 2MGF, 2MGG, 2MGH, 2MGI, 2MGJ, 2MGK, 2MGL, 2MGM, 2MYA, 2MYB, 2MYC, 2MYD, 2MYE, 2OH8, 2OH9, 2OHA, 2OHB, 2SPL, 2SPM, 2SPN, 2SPO, 2W6W, 2W6X, 2W6Y, 2Z6S, 2Z6T, 2ZSN, 2ZSO, 2ZSP, 2ZSQ, 2ZSR, 2ZSS, 2ZST, 2ZSX, 2ZSY, 2ZSZ, 2ZT0, 2ZT1, 2ZT2, 2ZT3, 2ZT4, 3A2G, 3ASE, 3E4N, 3E+55, 3E5I, 3E5O, 3ECL, 3ECX, 3ECZ, 3ED9, 3EDA, 3EDB, 3H57, 3H58, 3K9Z, 3M38, 3M39, 3M3A, 3M3B, 3MN0, 3NML, 3O89, 3OGB, 3SDN, 3U3E, 4FWX, 4FWY, 4FWZ, 4H07, 4H0B, 4IT8, 4LPI, 4MBN, 4MXK, 4MXL, 4NXA, 4NXC, 4OF9, 4PNJ, 4PQ6, 4PQB, 4PQC, 4QAU, 4TYX, 5B84, 5B85, 5C6Y, 5HAV, 5HLQ, 5HLU, 5HLX, 5JOM, 5IKS, 5ILE, 5ILM, 5ILP, 5ILR, 5KD1, 5KKK, 5M3S, 5MBN, 5O41, 5OJ9, 5OJA, 5OJB, 5OJC, 5UT7, 5UT8, 5UT9, 5UTA, 5UTB, 5UTC, 5UTD, 5VNU, 5VRT, 5VZN, 5VZO, 5VZP, 5WJK, 5XKV, 5XKW, 5XL0, 5YCE, 5YCH, 5YZF, 5ZEO, 5ZZF, 5ZZG, 6CF0, 6D45, 6F17, 6F18, 6F19, 6F1A, 6G5A, 6G5B, 6G5T, 6JP1, 6KRC, 6KRF, 6M8F, 6MV0, 6N02, 6N03, 6Z4R, 6Z4T, 7A44, 7A45, 7CEN, 7CEZ, 7EHX, 7KYR, 7L3U, 7L3Y	105M—crystallized in N-butyl isocyanide 1HJT—ferrous nitric oxide form. Excess NO was introduced into a closed vial containing the crystals 5VZQ**—**V68A/I107Y with nitric oxide. Numerous other mutants solved under the same conditions**—**only this one structure has the altered loop conformation 4OOD**—**K42Y, mutation adjacent to the HEME
P02186	*Elephas maximus* (Indian elephant)	1EMY	–
P02189	*Sus scrofa* (Pig)	1M6C, 1M6M, 1MDN, 1MNH, 1MNI, 1MNJ, 1MNK, 1MNO, 1MWC, 1MWD, 1MYG, 1MYH, 1MYI, 1PMB, 1YCA, 1YCB	–
P56208	*Caretta caretta* (Loggerhead sea turtle)	1LHS, 1LHT	–
P68082	*Equus caballus* (horse)	2IN4, 3BA2, 3V2V, 3V2Z—altered HEME 3HC9, 3HEN, 3HEO, 3HEP—common mutation to H64V adjacent to HEME 3RJN, 4NS2, 4TWU—common mutation pair D44K/D60K adjacent to HEME 6LTL, 6LTM, 3VM9, 3WYO, 7DGJ, 7DGK, 7DGL, 7DGM, 7DGN, 7DGO—dimeric, domain swapped, structures	1BJE, 1DWR, 1DWS, 1DWT, 1GJN, 1HRM, 1HSY, 1NPF, 1NPG, 1NZ2, 1NZ3, 1NZ4, 1NZ5, 1RSE, 1WLA, 1XCH, 1YMA, 1YMB, 1YMC, 2FRF, 2FRI, 2FRJ, 2FRK, 2NSR, 2NSS, 2O5B, 2O5L, 2O5M, 2O5O, 2O5Q, 2O5S, 2O5T, 2O58, 2V1E, 2V1F, 2V1G, 2V1H, 2V1I, 2V1J, 2V1K, 2VLX, 2VLY, 2VLZ, 2VM0, 3LR7, 3LR9, 3RJ6, 3VAU, 3WFT, 3WFU, 3WI8, 4DC7, 4DC8, 4TWV, 5AZQ, 5AZR, 5CMV, 5CN4, 5CN5, 5CN6, 5CN7, 5CN8, 5CN9, 5CNB, 5CNC, 5CND, 5CNE, 5CNF, 5CNG, 5D5R, 5YL3, 5Z7E, 5Z7F, 5ZZE
R9RZK8	*Balaena mysticetus* (Bowhead whale)	5YCI, 5YCJ	–
R9S002	*Mirounga angustirostris* (Northern elephant seal)	7DDU	–

*Myoglobin from *Thunnus thynnus* (Atlantic bluefin tuna), *Thunnus orientalis* (North Pacific bluefin tuna), *Thunnus albacares* (Yellowfin tuna) are excluded having shorter sequences and specifically in the loop between helices G and H which adopts are entirely different conformation as a result.

^#^Myoglobin from *Halichoerus grypus* (Gray seal) has a different conformation which cannot be confirmed since the structure is of lower resolution, 2.5 Å, and the density is not available in the PDB.

The identifying features of *Eq*_Myoglobin structures adopting the *Phy*_myoglobin are annotated in Table 1 and include (i) those having a wild type sequence but binding an altered heme cofactor (e.g. 3BA2, chlorin-substituted *Eq*_Myoglobin), (ii) those having certain mutations adjacent to the heme binding site, and (iii) heterodimeric, domain-swapped structures. The association with heme binding is curious given that the loop in question is located on the opposite end of the protein, at least 25 Å away, and in a loop not observed to have an altered conformation in the apo protein^4^. It was demonstrated decades ago that heme binding occurs cotranslationally^5^, and also that myoglobin can bind heme in different orientations having different rate constants^6^; more recently, molecular dynamics simulations showed that heme binding modulates the myoglobin folding pathway, increasing myoglobin stability and folding cooperativity^7^. Studies into the folding pathways of domain-swapped variants indicate differences between the native monomer and domain-swapped protein^8^. Together these observations could suggest that the variant loop conformations in *Eq_*myoglobin and *Phy_*myoglobin are products of subtly different folding pathways. Since synonymous coding has been suggested to alter folding pathways it is perhaps useful to compare the coding sequences of the horse and whale myoglobin proteins (Fig. 1E). Of note, the loop region bridging helices G and H contains synonymous mutations.

**Figure 2:**
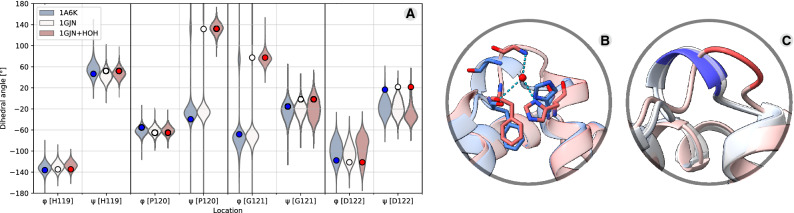
The horse heart turn conformation is associated with the presence of a structural water molecule. A display of dihedral angle distributions from 1us molecular dynamics simulations (violins) and initial values in PDB structures (circles) show that *Eq_*myoglobin (white) adopts the *Phy_*myoglobin (blue) turn conformation unless a structural water is maintained with the *Eq_*myoglobin (red) (**A**). This structural water forms three hydrogen bonds in the the *Eq_*myoglobin structure but not the *Phy_*myoglobin structure despite the presence of the same amino acid network (**B**). AlphaFold predicts *Phy_*myoglobin conformation from *Eq_*myoglobin sequence (grey cartoon, aligned to *Eq_*myoglobin 1GJN (red) and *Phy_*myoglobin 1A6K (blue)) (**C**).

To assess if these alternate loop orientations are intrinsically stable within the folded protein, we first ran molecular dynamics (MD) simulations on the myoglobin protein chain alone. In these simulations the initial *Eq_*myoglobin conformation quickly (10–20 ns) adjusted to and remained stable in the *Phy*_myoglobin conformation (refer to 1us simulations in Fig. 2A). Further analysis of the structures revealed a ubiquitously present water molecule, or electron density supporting water (e.g., 2VLY has a hydrogen peroxide molecule modelled in place of water, and 5AZQ has clear electron density in the 2Fo-Fc map despite no water molecule is modelled), bound in a network of three hydrogen bonds within the *Eq_*myoglobin loop conformation (Fig. 2B). MD simulations maintaining this structural water, demonstrated that its presence is sufficient to hold in place the alternate *Eq_*myoglobin loop conformation (Fig. 2A). This water molecule is not present in *Phy*_myoglobin structures or *Eq_*myoglobin having the *Phy*_myoglobin conformation, despite (spatial) conservation of the coordinating residues. Further supporting the critical role of this water in defining the local structure between helices G and H in *Eq_*myoglobin is AlphaFold2 structure prediction, which fails to account for sequence-extrinsic structural elements like water and predicts the *Eq_*myoglobin sequence in the *Phy*_myoglobin conformation (Fig. 2C).

## Discussion

We conclude that the distinct conformation adopted by the protein chain bridging helices G and H in *Phy*_myoglobin and *Eq*_myoglobin structures is consistent across a large ensemble of structures and cannot be readily explained by the sequence alone, the local environment of the protein chain, crystallization conditions or ambiguity in the structural models. When and how a water molecule, critical for maintaining the *Eq*_myoglobin turn conformation, becomes incorporated into the structure and how this is directed by mutations far removed from that location in sequence and space should be a matter for further investigations. The species specificity of this phenomenon, the observation that mutations affecting folding rate can alter the conformational preference and especially since turns are thought to be involved in the early stages of protein folding^9^ may implicate a protein folding mechanism. Our analysis suggests the continued usefulness of myoglobin as a “model” for protein folding, perhaps in the exploration of co-translational folding.

## Materials and methods

### Sequence alignment

Sequences were aligned using Clustalw^10^ and displayed using the ESPript 3.0 server^11^.

### Structure analysis

All structures associated with Uniprot^12^ entries for the *MB* gene were aligned and analysed using The PyMOL Molecular Graphics System, Version 2.0 Schrödinger, LLC^13^. Figures were prepared using ChimeraX^14^. Python 3.8 was utilized in displaying the molecular dynamics results and the final figures were crafted in Adobe Illustrator 2023.

### Molecular dynamics simulations

Simulations were run on apo-myoglobin using models 1GJN and 1A6K with all water molecules removed and additionally on 1GJN with water molecule 2112 held under position restraint with harmonic force constant of 100,000 kJ/mol^−1^ nm^−2^. All simulations were conducted using GROMACS software, version 2020.2^15^. The Amber 99sb-ildnp force field^16^ was applied to normal amino acids and ions, and the TIP3P model^17^ was applied to water molecules. After the energy minimizations and heating to 300 K, the system was equilibrated under NVT (constant volume and constant temperature) and NPT (constant pressure and constant temperature) conditions. Production runs were performed under NPT conditions, with a time step of 2 fs. The temperature and pressure were maintained at 300 K and 1 bar. Simulations were sampled every 20 ps for each trajectory and the distributions of each dihedral angle, for each model, were displayed on a violin plot. Two simulations with different initial velocities were conducted for each system to ensure reproducibility.

## Data Availability

The datasets (PDB and mtz files) analysed during the current study are freely available in the Protein Data Bank, https://www.rcsb.org/.
